# Lower rate of genomic variation identified in the trans-membrane domain of monoamine sub-class of Human G-Protein Coupled Receptors: The Human GPCR-DB Database

**DOI:** 10.1186/1471-2164-5-91

**Published:** 2004-12-04

**Authors:** Claes Wahlestedt, Anthony J Brookes, Salim Mottagui-Tabar

**Affiliations:** 1 Center for Genomics and Bioinformatics, Karolinska Institutet, Berzelius väg 35, 17177 Stockholm, Sweden; 2 Department of Genetics, University of Leicester, University Road, Leicester, LE1 7RH, UK

## Abstract

**Background:**

We have surveyed, compiled and annotated nucleotide variations in 338 human 7-transmembrane receptors (G-protein coupled receptors). In a sample of 32 chromosomes from a Nordic population, we attempted to determine the allele frequencies of 80 non-synonymous SNPs, and found 20 novel polymorphic markers. GPCR receptors of physiological and clinical importance were prioritized for statistical analysis. Natural variation and rare mutation information were merged and presented online in the Human GPCR-DB database .

**Results:**

The average number of SNPs per 1000 bases of exonic sequence was found to be twice the average number of SNPs per Kilobase of intronic regions (2.2 versus 1.0). Of the 338 genes, 111 were single exon genes, that is, were intronless. The average number of exonic-SNPs per single-exon gene was 3.5 (n = 395) while that for multi-exon genes was 0.8 (n = 1176). The average number of variations within the different protein domain (N-terminus, internal- and external-loops, trans-membrane region, C-terminus) indicates a lower rate of variation in the trans-membrane region of Monoamine GPCRs, as compared to Chemokine- and Peptide-receptor sub-classes of GPCRs.

**Conclusions:**

Single-exon GPCRs on average have approximately three times the number of SNPs as compared to GPCRs with introns. Among various functional classes of GPCRs, Monoamine GPRCs have lower number of natural variations within the trans-membrane domain indicating evolutionary selection against non-synonymous changes within the membrane-localizing domain of this sub-class of GPCRs.

## Background

The 7TM (7 trans-membrane domain proteins) genes, also known as the hetero-trimeric GTP-binding protein (G protein)-coupled receptors (GPCRs), are members of a large family of genes with an estimated 700 members in the human genome [1]. These receptors are plasma membrane-bound and have evolved to respond to a large number of extracellular and chemical signals. Upon interaction with their ligands, GPCRs act through the G proteins in signaling pathways that influence physiological functions. All GPCRs, in spite of great diversity in sequence composition, share a common protein structure. An N-terminal extracellular domain of variable length is followed by seven hydrophobic transmembrane-helices, connected by three intracellular (IL) and three extracellular (EL) loops, which then terminates in a C-terminal intracellular domain [2]. The functional and structural role of the different domains has been elucidated by systematic point mutations and crystal structure analysis for many of the human GPCR proteins. Several studies have collectively analyzed the occurrence, and importance of coding GPCR SNPs [3-5] and also the relevance and importance of mutations within these genes for the pharmaceutical industry [6]. The functional significance of thousands of point mutations has been described by a large number of investigations as evident at NCBI's PubMed Central. Mutation databases dedicated to GPCR mutations are currently available online like GPCR DB [7] and tinyGRAP [8]. Although an extensive collection of mutations is available at these sources, the distribution of these mutations and variations within the gene or peptide, along with common SNPs is not easily accessible or evident. The SNP databases in public domain (for example: NCBI's dbSNP) have highlighted all non-synonymous SNPs (nsSNPs). Also HGVBase  has further classified the location of the amino acid within the encoded proteins to more accurately predict the detrimental effects of a change in peptide sequence. From a pharmacogenetics viewpoint, the information about natural variations within GPCR transcripts and peptides, with allele frequency and validation data and disease association, is an important, yet currently unavailable, public resource. A database with functional promoter SNP, allele frequency, peptide variation information, population and haplotype information presented in a graphically accessible format would facilitate pharmacogenomics research related to GPCR proteins. HUMAN GPCR-DB aims to provide such a public resource. Also the rate of false positive SNPs determined experimentally in GPCR genes is reported to be relatively high [5]. For typical case-control association studies, prevailing designs favor highly polymorphic loci as against loci where the frequency of the minor allele is below 10%. Therefore more nsSNPs need to be validated and frequencies determined across ethnically diverse populations. We have designed genotyping assays and attempted to validate a number of GPCR nsSNPs for which no validation information was available on public databases, and deposited the validation information at HUMAN GPCR-DB. We have also collected published literature SNPs and added to our online database.

Several recent studies have focused on the subset of nsSNPs that most likely influence phenotype [9-13]. Comparatively, fewer attempts have been made on predicting and validating functional promoter SNPs [14]. As a part of a parallel work, we have developed a streamlined bioinformatics and wet-lab analysis methods to identify putative functional promoter SNPs with up to 70% probability of influencing gene expression. By applying this analysis package to all of the 338 genes in our database, we have highlighted, putative functional promoter SNPs. HUMAN GPCR-DB also attempts to merge SNP and other variations from published articles from PubMed and online SNP databases to facilitate direct identification of the functional significance of a natural variation.

## Results

A total of 427 non-redundant Human GPCR peptides were obtained from Swissprot of which 338 had Swissprot ID and the remaining had only TrEMBL identifications. Also, 89 of the 427 GPCR were classified as olfactory receptors. While all of the 427 entries were included in the HUMAN GPCR-DB database, only non-olfactory (i.e. 338) were considered for further analysis of gene structure, alternative transcripts, SNPs, and protein variations. Although the TrEMBL entries have also been included, the data displayed for these entries would be more accurately presented in future updates of the database. The statistical calculations for the genomic SNPs are based on the 338 non-olfactory entries, while the data for nsSNPs encoding peptide variations is based on 222 entries for which there are documented evidence for a 7-TM domain structure.

Transcript information for each gene was used for calculating average exonic and intronic SNPs. For genes with multiple transcript variants, the longest transcript was selected. Average number of SNPs per exon (n = 1511) of the 338 genes was one, while the average number of SNPs per intron (n = 1174) was nine. However, the average number of SNPs per 1000 bases of exonic sequence was twice the average number of SNPs per kilobase of intronic regions (2.2 versus 1.0). Of the 338 genes, 111 were single exon genes, that is, were intronless. The average number of exonic-SNPs per single-exon gene was 3.5 (n = 395) while that for multi-exon genes was 0.8 (n = 1176). This observation is in agreement with earlier observation based on a smaller number of GPCRs, where, compared to intronless GPCRs, exons in genes with introns on average had fewer SNPs [15].

Of the 1511 SNPs from the exons of 338 GPCR transcripts, 816 SNPs were coding SNPs, among which 392 were nsSNP; 211 of which had no validation information in either of the source databases, i.e. NCBI/dbSNP and HGVBase. The number of validated and non-validated SNPs is shown in Table 1. By excluding genes with 'probable', 'putative', 'precursor' and other ambiguous terms as a part of their description (as designated by Ensembl), we reduced the number of SNPs from 211 to 123 non-validated, nsSNPs which were considered as our list of prime candidates for the wet-lab validation process (Table 2). As a part of our first stage validation process, we designed assays for 80 of the 123 SNPs in GPCR genes of interest based on our better understanding of their role in human disease and physiology. Finally, of the 80 assayed SNPs, 20 were found to be polymorphic in our Nordic population sample consisting of DNA from 16 un-related healthy individuals. Of the 20 polymorphic markers 12 had a minor allele frequency higher than 10%.

**Table 1:** Distribution of validated and non-validated SNPs.

	Synonymous SNPs	Non-synonymous SNPs	Total SNPs
Validated SNPs	212	181	393
Non-validated SNPs	212	211	423
Total	424	392	816

The number of synonymous and non-synonymous SNPs and those with validation information, as deposited at NCBI/dbSNP, during 2003–4.

**Table 2:** Number of GPCRs in validated and non-validated categories.

Nr. of GPCR genes	Total nsSNPS	Validated nsSNPs	Non-validated nsSNPs	This study
338 (genes classified as GPCRs)	392	181	211 (123 were considered for validation).	80 of 123 assayed. 20 were polymorphic
222 (bearing evidence for 7-TM domains)	283	112 (Added 120 from [16])	171	
101 (classified in 3 sub-groups – Monoamine, Peptide and Chemokine).	182	53 (Added 83 from [16]) = 136 (used in Table 3).		

The initial 338 GPCRs were selected as per annotation by Ensembl. Support for structural evidence of 222 GPCRs was obtained from SWISSPROT. Classification of GPCRs was obtained from GPCRDB , Ensembl , International Union of Pharmacology .

Table 2 shows the number of GPCRs categorized by different criteria, and SNPs categorized in the two groups of validate and non-validated nsSNPs. Of the 338 genes, 222 had a documented GPCR structure as described by SWISSPROT/TrEMBL database. The total number of nsSNPs in this subset of 222 proteins was 283, of which 112 had validation information (leaving 171 with no validation information). To these we added 120 SNPs from Pubmed reports [14]. The identity of the 120 variations from published reports was verified to be SNPs and not rare mutations. Therefore a significant proportion of 'disease causing', rare variations were eliminated since they were reported from rare family based disease cases. These 120 SNPs are represented in the HUMAN GPCR-DB as 'rs-missing' since dbSNP records for many of these were not found. As future updates from dbSNP assign rs-IDs to the new SNPs, our database would update the records likewise.

According to the International Union of Pharmacology , 101 GPCRs from 338 were categorized either as 'peptide receptors' (n = 47) or 'chemokine receptors' (n = 54) or 'monoamine receptors' (n = 36). The distribution of nsSNPs across the 5 structural and functional domains (N-terminus, external loops, trans-membrane, internal loops and C-terminus) of these 101 GPCRs was calculated (Table 3). These 101 GPCRs have in total 182 nsSNPs, of which 53 SNPs have validation information in the major public databases. To these 53 SNPs we added 86 SNPs from published PubMed sources [14], bringing the total number of validated SNPs, used for this analysis, to 136 SNP. The 20 SNPs validated in this study were not included for this analysis since we wanted to analyze publicly available data only, at this time. The distribution of nsSNP numbers was compared between individual groups (monoamines-receptors only, or chemokine-receptors only or peptide-receptors only) and in various combinations with other two groups (monoamines plus chemokines or peptides plus monoamine, etc). None of the groups of receptors deviated from the mean of the three groups together, in any significant way. The N-terminus and external-loop SNPs were then combined in one group, and C-terminus and internal-loop SNPs in another group, and compared with nsSNPs in TM region. The nsSNP distribution in the three domains approached significance (Pearson's p-value 0.06) in the Monoamine sub-group of GPCRs. We then calculated the average number of nsSNP per 1000 bases of each of the 5 domains. The average number of nsSNPs in the TM region of Monoamine receptors (two SNPs per kilobase) was half of the average for each of the other groups (four or five nsSNPs per kilobase). This difference was not observed for any of the other four (N-term, e- and i-loops and C-term) structural domains of the peptides (Table 3).

**Table 3:** SNP distribution in peptide domains.

Peptide domain	Genes	N-term	e-loop	TM	i-loop	c-term	p-value	N-term + e-loop	TM	C-term + i-loop	p-value
Monoamine + Peptide + Chemokines	101	20 (3.7)	17 (3.6)	43 (3.0)	29 (3.5)	27 (4.5)		37 (3.7)	43 (3.0)	56 (4.0)	
Monoamine Only	36	7 (5.7)	6 (3.9)	11 (1.9)	16 (3.3)	18 (3.9)	0.26	13 (4.7)	11 (1.9)	34 (3.5)	0.06
Peptide Only	47	10 (2.9)	8 (3.9)	25 (4.2)	11 (4.3)	15 (5.7)	0.89	18 (3.3)	25 (4.2)	26 (5.0)	0.79
Chemokine Only	18	3 (4.0)	3 (2.8)	7 (2.5)	2 (2.5)	4 (4.5)	0.87	6 (3.3)	7 (2.5)	6 (3.5)	0.72
Chemokine + Monoamine	54	10 (5.1)	9 (3.4)	18 (2.1)	18 (3.2)	12 (4.1)	0.93	19 (4.1)	18 (2.1)	30 (3.5)	0.78
Peptide + Monoamine	83	17 (3.7)	14 (3.9)	36 (3.2)	27 (3.6)	23 (4.9)	0.94	31 (3.8)	36 (3.2)	50 (4.2)	0.96
Chemokine + Peptide	65	13 (3.1)	11 (3.5)	32 (3.7)	13 (3.8)	19 (5.4)	0.87	24 (3.3)	32 (3.7)	32 (4.6)	0.71

Total number of nsSNP in various domains of 3 subgroups of GPCR proteins.

Abbreviations: N-term : N terminus; C-term : C terminus; i-loop : internal loops; e-loop : external loops; TM: trans-membrane. The numbers in the brackets are the average number of SNPs per 1000 base pairs of a specific domain. The Fisher's Exact p-values were calculated using a 5 × 2 contingency table at . Contingency tables of 2 × 3 were constructed at . P-values of below or close to 0.05 are considered significant.

We compiled together the functional properties of peptide variations from published records along with the knowledge of the location and the two alleles of a nsSNP in our database. Searching PubMed records, we found 38 nsSNPs located in the precise position, and substituting the same amino acid, as those studied for functional analysis shown in Table 4 [See additional file 1].

### Database interface and layout

The HUMAN GPCR-DB is currently online . This database allows for 3 alternative queries, either Ensembl gene ID, or Swissprot/TrEMBL ID or part of the gene name or description. Resulting hits are displayed along with total number of coding SNPs and protein variations. These links in turn display a graphic representation of the SNP locations within exons (or peptide) and the query gene. The exons are drawn in proportion to the largest exon, while the introns are of fixed length. The SNP list provides allele information, flanking sequences (for assay development and strand verification, etc) and links for validation information and source databases. The promoter information is drawn in a similar manner, with mouse conserved regions indicated with green bars underneath and SNPs within conserved regions marked with a symbol 'M' in the SNP full-list. SNPs predicted to influence protein binding according to our prediction model are marked 'T' in the SNP full-list. The link for protein variations displays a window with SNP, marked in red arrows, and mutation distribution across 3 regions of the peptide; N-terminus, C-terminus and trans-membrane and loop regions. Association with diseases and the corresponding PubMed ID are displayed as a popup menu following the link under 'disease' column.

## Discussion

We have constructed a database, which combines mutation information with validated SNP information from publicly available sources. We have then attempted to validate and determine the frequencies of 80 of the 123 non-synonymous SNPs for which no validation information was available publicly. Proportion of true polymorphic loci was 20%, in agreement with reported expectations from several studies [5].

The statistical approach for the analysis of distribution of natural variations in GPCRs, presented here is borrowed from two recent studies [15,16]. While in the first of these studies [15] 64 GPCR genes were sequenced in 82 individuals of divergent ethnic backgrounds and resulting frequency distribution of nsSNPs were compared with non-GPCR genes, the later study [16] analyzed differences in distribution of published and publicly available nsSNP in 62 GPCR genes, across the 5 peptide domains. For our current report we analyzed 222 GPCR genes with over 200 nsSNPs (283 snSNPs available on public databases, and 120 nsSNP from PubMed records) [16]. Transcripts lacking introns had on average higher density of SNPs (2-fold) than those with introns, in agreement with an earlier published report [15]. The distribution of the nsSNP in the 5 different peptide domains (N-term, e-loops, trans-membrane, i-loops and C-term) was found not to be different between any of the ligand specific sub-groups of GPCR proteins, namely peptide receptors, monoamine receptors and chemokine receptors. We reasoned that the evolutionary constraints on outer- and inner-loops along with the N-term and C-term regions would be related to the function of these regions while the constraints on the trans-membrane regions might be related to their structure. We, therefore compared the distribution of validated nsSNP in the 3 major peptide domains (e-loops + N-term = region 1; trans-membrane = region 2; i-loops + C-term = region 3). We observed a difference in nsSNP distribution across the three regions, which approached significance (Pearson's p-value = 0.06). There was a two-fold decrease in frequency of occurrence of nsSNP in the TM region of monoamine receptors as compared to the other sub-groups of receptors. This indicates that there might perhaps be a functional selection against variations, acting on TM domains of monoamine receptors, which is less selective on the TM domains of peptide receptors, and chemokine receptor GPCRs. A recent study reported differences between the distribution of 'disease causing' and 'non-disease causing' variations in different sub-groups of GPCR family members [16]. Our study excluded rare mutations, which were known to be associated with disease, and therefore a similar comparison was not possible.

Although HUMAN GPCR-DB database does obtain the bulk of the information and data from Ensembl and dbSNP, it is not merely a subset of these major databases. While Ensembl provides sequence and genetic variation information, it provides SNP validation information obtained from public sources, which may include, as shown in several published studies, up to 50% false positives. NCBI's dbSNP provides validation-, submitter- and method-information, yet rates of false positives have proven to be high. These databases harbor information of genetic variations for all coding and non-coding regions of the human genome. The HUMAN GPCR-DB, in addition to providing this set of information, provides in-house validation and assay-information for non-validated nsSNPs. HUMAN GPCR-DB provides natural variation, mutation, promoter- and peptide-variation information along with gene structure and peptide 7-TM structure information and SNP validation information of a focused group of clinically important genes.

Transcriptional regulatory regions on the 5'-FR of human genes encode short sequences which serve as targets for binding of transcription factors (TFs). Eukaryotic TFs tolerate considerable sequence variation in their target sites and recent works in bioinformatics [17-19] have developed reliable methods to model the DNA binding specificity of individual TFs [20]. Currently the most successful approach to overcome this information gap is based on the assumption that gene sequences conserved between species (here Human and Mouse) would most likely mediate biological function [21-25]. Our recent study (our manuscript, 2004) describes a method for the detection and validation of functionally important SNPs in the 5'-flanking regions of human. The rate of successful detection of SNPs influencing TFBS using our method is approximately 70%. This prediction algorithm has been used to highlight SNPs in 5' flanking regions of the GPCR in the HUMAN GPCR-DB genes to facilitate selection and study of functionally important promoter SNPs. The knowledge of functional promoter SNPs would help us study disease related GPCR in more details.

The functional domains of human GPCRs have over the passed decade been dissected by systematically mutating the peptide sequence [8]. A collection of mutations and their disease significance and influence on the function of the protein together with common variations within the human population would facilitate our understanding of the variations and disease association. HUMAN GPCR-DB attempts to merge SNP and mutation information along with disease information in easily accessible and user-friendly manner. Although direct links to disease databases would be included in the next release, the existing information about the source publication can be helpful in obtaining the relevant details about the mutations.

### Current and future updates

Of the 123 nsSNPs without validation information, we have currently validated 80 SNPs. Future updates would include information about the remaining nsSNPs, and any additional which are reported by public databases. We have also collected 120 published SNPs, which have as yet not been deposited at public databases, or are in the process of being deposited. We would have a complete update of SNP data for every new release of NCBI and HGVBase SNP tables. New GPCR identification and characterization, or changes in existing GPCR genes or proteins information would be updated once a year from Ensembl and SwissProt Databases. PubMed references would be updated monthly or as often as necessary to complete the mutation coverage of the 222 GPCR proteins. Haplotype information and genetic association studies for available SNPs along with published records on functional promoter SNPs would be added. A valuable addition would be to indicate variation frequencies in ethnically diverse populations. We are currently adding such information about the allele frequencies and populations in the database and would be provided in future updates.

## Conclusions

Single-exon GPCRs on average have approximately three times the number of SNPs as compared to GPCRs with introns. Among various functional classes of GPCRs, Monoamine GPRCs have lower number of natural variations within the trans-membrane domain indicating evolutionary selection against non-synonymous changes within membrane localizing domain. The HUMAN GPCR-DB compiles SNPs and mutations in one database. Using a recently developed method for identification of functionally important SNPs in the 5'-flanking regions of human, with approximately 70% success rate, the database highlights such SNPs to facilitate selection and study of functionally important promoter SNPs.

## Methods

The list of Human GPCR genes was compiled by collecting gene names from several different sources and subsequently the list was updated by removing duplicates and entries with incomplete information like peptide fragments, partial sequences and hypothetical proteins. The Ensembl MART genome server database was queried for GPCR family members. The Gene Ontology server Amigo  was queried with the search term 'GO:0004930', which describes the GPCR group of genes. The list of Swiss-Prot and TrEMBL entries were fetched from GPCRDB [7], ensemble, International Union of Pharmacology . All the lists were merged and redundancies removed and Ensembl gene numbers (ENSG) were obtained for all of the genes from Ensembl genome server. The final list consisted of total of 427 genes with unique ENSG numbers. Of these 427 genes, 89 genes belonged to the sub-family of olfactory genes. Also of the 427 genes, 222 had demonstrable or convincing evidence for GPCR domain structure and sequence information in Ensembl and SwissProt databases.

The gene and transcript map information were obtained from Ensembl databases 'homo_sapien_core_25_34d' and 'ensemble_mart_25_1', released in September 2004. The tables for gene mapping, SNP mapping and Human-Mouse alignment were obtained from ensemble, dbSNP, HGVBASE and UCSC and installed locally. For the chromosomal and genomic location of SNPs and validation information, NCBI's dbSNP tables 'snp', 'snpcontigloc', and 'snpcontiglocusid' and Ensembl's tables 'ContigHit' and 'locus' were used. Information about the location of the SNP within protein domains was obtained from Swissprot using bioperl modules for accessing protein features. HGVBase release version 14 was used for obtaining HGVBASE SNP identities and validation and frequency information. The flanking sequence information was obtained from both Ensembl's RefSNP table and by downloading sequence flat files from dSNP (ds_flat_chr'1–22. X, Y'.fa);

For mapping Transcription Factor Binding Sites, the TFBS perl programming system [26] was used. This program applies position weight matrices (PWM) to DNA sequences to generate mathematical probability for the binding of a TF, based on the earlier described thermodynamics of binding energy [27-29]. Recent reviews and articles describe methods related to PWM and the bioinformatics of regulatory site prediction [18,30]. For determining human-mouse conserved regions, global best alignments files were downloaded from UCSC. SNPs in 5' flanking sequences were analyzed according to the differences in the absolute bind score derived from the matrices for each TF. A total of 78 factors from vertebrate class were used, hosted at the TFBS database, JASPAR [31]. MySQL™ version 4.0 with ActiveState™ Komodo version 2.3 as perl programming IDE and bioperl modules version 1.2 were used for database development. The web server technology used was Apache™ 2.0 with PHP 4.0, as supplied by NuSphere™ version 3.0. Non-synonymous SNPs were identified from all the SNPs, after the construction of the GPCR SNP database based on data acquired directly from dbSNP and HGVBASE. Validation of SNPs was carried out by DynaMetrix Inc., UK, using the DASH platform [32]. The allele frequency validation was performed on DNA samples from 16 anonymous individuals of Nordic descent, with the Institutional Review Board Approval KI 02-544.

## Abbreviations

7TM: 7 transmembrane; GPCR:G-protein Coupled Receptors; SNP: single Nucleotide Polymorphism; nsSNP : non-synonymous SNPs; cSNP : coding SNP; rSNP : regulatory SNPs; N-term : N terminus; C-term : C terminus; e-loop : external loops; i-loop : internal loops.

## Authors' contributions

SM-T did all the coding, analysis, manuscript preparation and reviewer correspondence. AJB contributed genotyping information and validated a number of SNPs. CW provided running costs and assistance with writing the manuscript. All authors read and approved the final manuscript.

## Supplementary Material

Additional File 1 Table 4 A list of natural non-synonymous variations and mutations with references to articles describing the phenotype associated with the variation. Click here for file
