# Supplementary material for: Lower rate of genomic variation identified in the trans-membrane domain of monoamine sub-class of Human G-Protein Coupled Receptors: The Human GPCR-DB Database
Source: BMC Genomics. 2004 Dec 4;5:91. doi: 10.1186/1471-2164-5-91 (PMC538281; doi:10.1186/1471-2164-5-91)
Supplement: Additional File 1 — Table 4 A list of natural non-synonymous variations and mutations with references to articles describing the phenotype associated with the variation. [file 1471-2164-5-91-S1.doc]

Table 4

SNPs and Mutations

| SwissProt Name | Mutation | SNP ID | SNP | Region | Phenotype | Reference |
| --- | --- | --- | --- | --- | --- | --- |
|  |  |  |  |  |  |  |
| 5H1B_HUMAN | F124C | rs130060 | C124F | TRANSMEM_3 |  | [1] |
| 5H2C_HUMAN | C23S | rs6318 | S23C | N_term | Obesity, anorexia | [2] |
| CASR_HUMAN | A986S | rs1801725 | S986A | C_term | Hypercalciuria | [3,4] |
| CKR2_HUMAN | V64I | rs1799864 | I64V | TRANSMEM_1 |  | [5,6] |
| CKR5_HUMAN | C101A | rs1800560 | C101C | LOOP_2 |  | [7-9] |
| CKR5_HUMAN | R223Q | rs1800452 | Q223R | LOOP_5 |  | [10] |
| D2DR_HUMAN | P310S | rs1800496 | S310P | LOOP_5 | Schizophrenia | [11] |
| D2DR_HUMAN | S311C | rs1801028 | C311S | LOOP_5 | Alcoholism, Schizophrenia | [12,13] |
| D3DR_HUMAN | S9G | rs6280 | S9G | N_term | Schizophrenia | [14] |
| DBDR_HUMAN | L88F | rs6282 | R88L | TRANSMEM_2 | Psychiatric disorders | [15,16] |
| DBDR_HUMAN | A269V | rs2227842 | V269A | LOOP_5 | Psychiatric disorders | [17] |
| ETBR_HUMAN | S305N | rs5352 | N305S | LOOP_5 | Hirschsprung | [18,19] |
| FSHR_HUMAN | T307A | rs6165 | T307A | N_term | Familial twinning | [20-22] |
| FSHR_HUMAN | N680S | rs6166 | N680S | C_term | Polycystic Ovary Syndrome | [23-25] |
| GALR_HUMAN | C15W | rs1143093 | C15W | N_term |  | [26] |
| GBR1_HUMAN | G489S | rs1805057 | S489G | N_term | Schizophrenia | [27,28] |
| LSHR_HUMAN | S312N | rs2293275 | S312N | N_term | Pseudohermaphroditism | [29,30] |
| MSHR_HUMAN | V60L | rs1805005 | L60V | TRANSMEM_1 | Red hair | [31] |
| MSHR_HUMAN | V92M | rs2228479 | M92V | TRANSMEM_2 |  | [32,33] |
| MSHR_HUMAN | R151C | rs1805007 | C151R | LOOP_3 |  | [34,35] |
| MSHR_HUMAN | R160W | rs1805008 | W160R | LOOP_3 |  | [36] |
| MSHR_HUMAN | F196A | rs3212366 | L196F | TRANSMEM_5 |  | [37] |
| MSHR_HUMAN | D294H | rs1805009 | H294D | TRANSMEM_7 |  | [38,39] |
| OPRM_HUMAN | N40D | rs1799971 | D40N | N_term | Alcohol Dependency | [40-42] |
| OXYR_HUMAN | N57A | rs2228485 | N57N | TRANSMEM_1 |  | [43,44] |
| PAFR_HUMAN | A224D | rs5938 | D224A | LOOP_5 |  | [45] |
| PAR2_HUMAN | N30A | rs616235 | N30S | SIGNAL |  | [46] |
| PI2R_HUMAN | V25M | rs2229127 | M25V | TRANSMEM_1 |  | [47,48] |
| PI2R_HUMAN | R212H | rs2229131 | H212R | LOOP_5 |  | [49,50] |
| PI2R_HUMAN | S328A | rs2229129 | S328S | C_term |  | [51-53] |
| SSR5_HUMAN | P335L | rs169068 | L335P | C_term | Bipolar affective disorder | [54] |
| TSHR_HUMAN | P52T | rs2234919 | T52P | N_term | Hypothroidism | [55-57] |
| TSHR_HUMAN | D727E | rs1991517 | D727E | C_term | Multinodular Goiter | [58-60] |
| V2R_HUMAN | T7S | rs5196 | S7T | N_term | Nephrogenic Diabetes | [61] |
| V2R_HUMAN | G12E | rs2071126 | E12G | N_term |  | [62] |
| V2R_HUMAN | A147T | rs5200 | V147A | LOOP_3 | Nephrogenic Diabetes | [63-65] |
| V2R_HUMAN | L309P | rs5201 | L309L | TRANSMEM_7 | Nephrogenic Diabetes | [66] |
| V2R_HUMAN | S371A | rs5203 | S371S | C_term |  | [67] |

Legend: Mutation and SNPs investigated in association to human phenotype.

Reference List

1. Sanders AR, Duan J, Gejman PV: **DNA variation and psychopharmacology of the human serotonin receptor 1B (HTR1B) gene.** *Pharmacogenomics* 2002, **3:** 745-762.

2. Lentes KU, Hinney A, Ziegler A, Rosenkranz K, Wurmser H, Barth N, Jacob K, Coners H, Mayer H, Grzeschik KH, Schafer H, Remschmidt H, Pirke KM, Hebebrand J: **Evaluation of a Cys23Ser mutation within the human 5-HT2C receptor gene: no evidence for an association of the mutant allele with obesity or underweight in children, adolescents and young adults.** *Life Sci* 1997, **61:** L9-16.

3. Eckstein M, Vered I, Ish-Shalom S, Shlomo AB, Shtriker A, Koren-Morag N, Friedman E: **Vitamin D and calcium-sensing receptor genotypes in men and premenopausal women with low bone mineral density.** *Isr Med Assoc J* 2002, **4:** 340-344.

4. Lerolle N, Coulet F, Lantz B, Paillard F, Houillier P, Soubrier F, Gattegno B, Jeunemaitre X, Ronco P, Rondeau E: **No evidence for point mutations of the calcium-sensing receptor in familial idiopathic hypercalciuria.** *Nephrol Dial Transplant* 2001, **16:** 2317-2322.

5. Kostrikis LG, Huang Y, Moore JP, Wolinsky SM, Zhang L, Guo Y, Deutsch L, Phair J, Neumann AU, Ho DD: **A chemokine receptor CCR2 allele delays HIV-1 disease progression and is associated with a CCR5 promoter mutation.** *Nat Med* 1998, **4:** 350-353.

6. Petersen DC, Laten A, Zeier MD, Grimwood A, Rensburg EJ, Hayes VM: **Novel mutations and SNPs identified in CCR2 using a new comprehensive denaturing gradient gel electrophoresis assay.** *Hum Mutat* 2002, **20:** 253-259.

7. Blanpain C, Lee B, Vakili J, Doranz BJ, Govaerts C, Migeotte I, Sharron M, Dupriez V, Vassart G, Doms RW, Parmentier M: **Extracellular cysteines of CCR5 are required for chemokine binding, but dispensable for HIV-1 coreceptor activity.** *J Biol Chem* 1999, **274:** 18902-18908.

8. Chelli M, Alizon M: **Determinants of the trans-dominant negative effect of truncated forms of the CCR5 chemokine receptor.** *J Biol Chem* 2001, **276:** 46975-46982.

9. Genoud S, Kajumo F, Guo Y, Thompson D, Dragic T: **CCR5-Mediated human immunodeficiency virus entry depends on an amino-terminal gp120-binding site and on the conformational integrity of all four extracellular domains.** *J Virol* 1999, **73:** 1645-1648.

10. Blanpain C, Lee B, Vakili J, Doranz BJ, Govaerts C, Migeotte I, Sharron M, Dupriez V, Vassart G, Doms RW, Parmentier M: **Extracellular cysteines of CCR5 are required for chemokine binding, but dispensable for HIV-1 coreceptor activity.** *J Biol Chem* 1999, **274:** 18902-18908.

11. Duan J, Wainwright MS, Comeron JM, Saitou N, Sanders AR, Gelernter J, Gejman PV: **Synonymous mutations in the human dopamine receptor D2 (DRD2) affect mRNA stability and synthesis of the receptor.** *Hum Mol Genet* 2003, **12:** 205-216.

12. Duan J, Wainwright MS, Comeron JM, Saitou N, Sanders AR, Gelernter J, Gejman PV: **Synonymous mutations in the human dopamine receptor D2 (DRD2) affect mRNA stability and synthesis of the receptor.** *Hum Mol Genet* 2003, **12:** 205-216.

13. Goldman D, Urbanek M, Guenther D, Robin R, Long JC: **Linkage and association of a functional DRD2 variant [Ser311Cys] and DRD2 markers to alcoholism, substance abuse and schizophrenia in Southwestern American Indians.** *Am J Med Genet* 1997, **74:** 386-394.

14. Lundstrom K, Turpin MP: **Proposed schizophrenia-related gene polymorphism: expression of the Ser9Gly mutant human dopamine D3 receptor with the Semliki Forest virus system.** *Biochem Biophys Res Commun* 1996, **225:** 1068-1072.

15. Cravchik A, Gejman PV: **Functional analysis of the human D5 dopamine receptor missense and nonsense variants: differences in dopamine binding affinities.** *Pharmacogenetics* 1999, **9:** 199-206.

16. Feng J, Sobell JL, Heston LL, Cook EH, Jr., Goldman D, Sommer SS: **Scanning of the dopamine D1 and D5 receptor genes by REF in neuropsychiatric patients reveals a novel missense change at a highly conserved amino acid.** *Am J Med Genet* 1998, **81:** 172-178.

17. Cravchik A, Gejman PV: **Functional analysis of the human D5 dopamine receptor missense and nonsense variants: differences in dopamine binding affinities.** *Pharmacogenetics* 1999, **9:** 199-206.

18. Abe Y, Sakurai T, Yamada T, Nakamura T, Yanagisawa M, Goto K: **Functional analysis of five endothelin-B receptor mutations found in human Hirschsprung disease patients.** *Biochem Biophys Res Commun* 2000, **275:** 524-531.

19. Auricchio A, Casari G, Staiano A, Ballabio A: **Endothelin-B receptor mutations in patients with isolated Hirschsprung disease from a non-inbred population.** *Hum Mol Genet* 1996, **5:** 351-354.

20. Al Hendy A, Moshynska O, Saxena A, Feyles V: **Association between mutations of the follicle-stimulating-hormone receptor and repeated twinning.** *Lancet* 2000, **356:** 914.

21. Montgomery GW, Duffy DL, Hall J, Kudo M, Martin NG, Hsueh AJ: **Mutations in the follicle-stimulating hormone receptor and familial dizygotic twinning.** *Lancet* 2001, **357:** 773-774.

22. Tong Y, Liao WX, Roy AC, Ng SC: **Association of AccI polymorphism in the follicle-stimulating hormone beta gene with polycystic ovary syndrome.** *Fertil Steril* 2000, **74:** 1233-1236.

23. Al Hendy A, Moshynska O, Saxena A, Feyles V: **Association between mutations of the follicle-stimulating-hormone receptor and repeated twinning.** *Lancet* 2000, **356:** 914.

24. Montgomery GW, Duffy DL, Hall J, Kudo M, Martin NG, Hsueh AJ: **Mutations in the follicle-stimulating hormone receptor and familial dizygotic twinning.** *Lancet* 2001, **357:** 773-774.

25. Simoni M, Gromoll J, Hoppner W, Kamischke A, Krafft T, Stahle D, Nieschlag E: **Mutational analysis of the follicle-stimulating hormone (FSH) receptor in normal and infertile men: identification and characterization of two discrete FSH receptor isoforms.** *J Clin Endocrinol Metab* 1999, **84:** 751-755.

26. Sullivan KA, Shiao LL, Cascieri MA: **Pharmacological characterization and tissue distribution of the human and rat GALR1 receptors.** *Biochem Biophys Res Commun* 1997, **233:** 823-828.

27. Hisama FM, Gruen JR, Choi J, Huseinovic M, Grigorenko EL, Pauls D, Mattson RH, Gelernter J, Wood FB, Goei VL: **Human GABA(B) receptor 1 gene: eight novel sequence variants.** *Hum Mutat* 2001, **17:** 349-350.

28. Imai K, Harada S, Kawanishi Y, Tachikawa H, Okubo T, Asada T: **Association analysis of an (AC)n repeat polymorphism in the GABA(B) receptor gene and schizophrenia.** *Am J Med Genet* 2002, **114:** 605-608.

29. Laue L, Wu SM, Kudo M, Hsueh AJ, Cutler GB Jr, Jelly DH, Diamond FB, Chan WY: **Heterogeneity of activating mutations of the human luteinizing hormone receptor in male-limited precocious puberty.** *Biochem Mol Med* 1996, **58:** 192-198.

30. Misrahi M, Meduri G, Pissard S, Bouvattier C, Beau I, Loosfelt H: **Comparison of immunocytochemical and molecular features with the phenotype in a case of incomplete male pseudohermaphroditism associated with a mutation of the luteinizing hormone receptor.** *J Clin Endocrinol Metab* 1997, **82:** 2159-2165.

31. Schioth HB, Phillips SR, Rudzish R, Birch-Machin MA, Wikberg JE, Rees JL: **Loss of function mutations of the human melanocortin 1 receptor are common and are associated with red hair.** *Biochem Biophys Res Commun* 1999, **260:** 488-491.

32. Koppula SV, Robbins LS, Lu D, Baack E, White CR, Jr., Swanson NA: **Identification of common polymorphisms in the coding sequence of the human MSH receptor (MCIR) with possible biological effects.** *Hum Mutat* 1997, **9:** 30-36.

33. Prusis P, Schioth HB, Muceniece R, Herzyk P, Afshar M, Hubbard RE: **Modeling of the three-dimensional structure of the human melanocortin 1 receptor, using an automated method and docking of a rigid cyclic melanocyte-stimulating hormone core peptide.** *J Mol Graph Model* 1997, **15:** 307-17, 334.

34. Frandberg PA, Doufexis M, Kapas S, Chhajlani V: **Amino acid residues in third intracellular loop of melanocortin 1 receptor are involved in G-protein coupling.** *Biochem Mol Biol Int* 1998, **46:** 913-922.

35. Schioth HB, Phillips SR, Rudzish R, Birch-Machin MA, Wikberg JE, Rees JL: **Loss of function mutations of the human melanocortin 1 receptor are common and are associated with red hair.** *Biochem Biophys Res Commun* 1999, **260:** 488-491.

36. Schioth HB, Phillips SR, Rudzish R, Birch-Machin MA, Wikberg JE, Rees JL: **Loss of function mutations of the human melanocortin 1 receptor are common and are associated with red hair.** *Biochem Biophys Res Commun* 1999, **260:** 488-491.

37. Yang YK, Ollmann MM, Wilson BD, Dickinson C, Yamada T, Barsh GS: **Effects of recombinant agouti-signaling protein on melanocortin action.** *Mol Endocrinol* 1997, **11:** 274-280.

38. Prusis P, Schioth HB, Muceniece R, Herzyk P, Afshar M, Hubbard RE: **Modeling of the three-dimensional structure of the human melanocortin 1 receptor, using an automated method and docking of a rigid cyclic melanocyte-stimulating hormone core peptide.** *J Mol Graph Model* 1997, **15:** 307-17, 334.

39. Schioth HB, Phillips SR, Rudzish R, Birch-Machin MA, Wikberg JE, Rees JL: **Loss of function mutations of the human melanocortin 1 receptor are common and are associated with red hair.** *Biochem Biophys Res Commun* 1999, **260:** 488-491.

40. Bergen AW, Kokoszka J, Peterson R, Long JC, Virkkunen M, Linnoila M: **Mu opioid receptor gene variants: lack of association with alcohol dependence.** *Mol Psychiatry* 1997, **2:** 490-494.

41. Bond C, LaForge KS, Tian M, Melia D, Zhang S, Borg L: **Single-nucleotide polymorphism in the human mu opioid receptor gene alters beta-endorphin binding and activity: possible implications for opiate addiction.** *Proc Natl Acad Sci U S A* 1998, **95:** 9608-9613.

42. Wand GS, McCaul M, Yang X, Reynolds J, Gotjen D, Lee S: **The mu-opioid receptor gene polymorphism (A118G) alters HPA axis activation induced by opioid receptor blockade.** *Neuropsychopharmacology* 2002, **26:** 106-114.

43. Fanelli F, Barbier P, Zanchetta D, de Benedetti PG, Chini B: **Activation mechanism of human oxytocin receptor: a combined study of experimental and computer-simulated mutagenesis.** *Mol Pharmacol* 1999, **56:** 214-225.

44. Gimpl G, Fahrenholz F: **The oxytocin receptor system: structure, function, and regulation.** *Physiol Rev* 2001, **81:** 629-683.

45. Fukunaga K, Ishii S, Asano K, Yokomizo T, Shiomi T, Shimizu T: **Single nucleotide polymorphism of human platelet-activating factor receptor impairs G-protein activation.** *J Biol Chem* 2001, **276:** 43025-43030.

46. Compton SJ, Sandhu S, Wijesuriya SJ, Hollenberg MD: **Glycosylation of human proteinase-activated receptor-2 (hPAR2): role in cell surface expression and signalling.** *Biochem J* 2002, **368:** 495-505.

47. Stitham J, Stojanovic A, Hwa J: **Impaired receptor binding and activation associated with a human prostacyclin receptor polymorphism.** *J Biol Chem* 2002, **277:** 15439-15444.

48. Stitham J, Stojanovic A, Merenick BL, O'Hara KA, Hwa J: **The unique ligand-binding pocket for the human prostacyclin receptor. Site-directed mutagenesis and molecular modeling.** *J Biol Chem* 2003, **278:** 4250-4257.

49. Stitham J, Stojanovic A, Hwa J: **Impaired receptor binding and activation associated with a human prostacyclin receptor polymorphism.** *J Biol Chem* 2002, **277:** 15439-15444.

50. Stitham J, Stojanovic A, Merenick BL, O'Hara KA, Hwa J: **The unique ligand-binding pocket for the human prostacyclin receptor. Site-directed mutagenesis and molecular modeling.** *J Biol Chem* 2003, **278:** 4250-4257.

51. Miggin SM, Kinsella BT: **Investigation of the mechanisms of G protein: effector coupling by the human and mouse prostacyclin receptors. Identification of critical species-dependent differences.** *J Biol Chem* 2002, **277:** 27053-27064.

52. Smyth EM, Li WH, FitzGerald GA: **Phosphorylation of the prostacyclin receptor during homologous desensitization. A critical role for protein kinase c.** *J Biol Chem* 1998, **273:** 23258-23266.

53. Smyth EM, Austin SC, Reilly MP, FitzGerald GA: **Internalization and sequestration of the human prostacyclin receptor.** *J Biol Chem* 2000, **275:** 32037-32045.

54. Nyegaard M, Borglum AD, Bruun TG, Collier DA, Russ C, Mors O: **Novel polymorphisms in the somatostatin receptor 5 (SSTR5) gene associated with bipolar affective disorder.** *Mol Psychiatry* 2002, **7:** 745-754.

55. Loos U, Hagner S, Bohr UR, Bogatkewitsch GS, Jakobs KH, Van Koppen CJ: **Enhanced cAMP accumulation by the human thyrotropin receptor variant with the Pro52Thr substitution in the extracellular domain.** *Eur J Biochem* 1995, **232:** 62-65.

56. Simanainen J, Kinch A, Westermark K, Winsa B, Bengtsson M, Schuppert F *et al*.: **Analysis of mutations in exon 1 of the human thyrotropin receptor gene: high frequency of the D36H and P52T polymorphic variants.** *Thyroid* 1999, **9:** 7-11.

57. Tonacchera M, Cetani F, Costagliola S, Van Sande J, Refetoff S, Vassart G: **Functional characteristics of a variant thyrotropin receptor.** *Eur J Biochem* 1996, **238:** 490-494.

58. Gabriel EM, Bergert ER, Grant CS, van Heerden JA, Thompson GB, Morris JC: **Germline polymorphism of codon 727 of human thyroid-stimulating hormone receptor is associated with toxic multinodular goiter.** *J Clin Endocrinol Metab* 1999, **84:** 3328-3335.

59. Muhlberg T, Herrmann K, Joba W, Kirchberger M, Heberling HJ, Heufelder AE: **Lack of association of nonautoimmune hyperfunctioning thyroid disorders and a germline polymorphism of codon 727 of the human thyrotropin receptor in a European Caucasian population.** *J Clin Endocrinol Metab* 2000, **85:** 2640-2643.

60. Nogueira CR, Nguyen LQ, Coelho-Neto JR, Arseven OK, Jameson JL, Kopp P: **Structural analysis of the thyrotropin receptor in four patients with congenital hypothyroidism due to thyroid hypoplasia.** *Thyroid* 1999, **9:** 523-529.

61. Arthus MF, Lonergan M, Crumley MJ, Naumova AK, Morin D, De Marco LA: **Report of 33 novel AVPR2 mutations and analysis of 117 families with X-linked nephrogenic diabetes insipidus.** *J Am Soc Nephrol* 2000, **11:** 1044-1054.

62. Wenkert D, Schoneberg T, Merendino JJ, Jr., Rodriguez Pena MS, Vinitsky R, Goldsmith PK: **Functional characterization of five V2 vasopressin receptor gene mutations.** *Mol Cell Endocrinol* 1996, **124:** 43-50.

63. Erlenbach I, Kostenis E, Schmidt C, Serradeil-Le Gal C, Raufaste D, Dumont ME: **Single amino acid substitutions and deletions that alter the G protein coupling properties of the V2 vasopressin receptor identified in yeast by receptor random mutagenesis.** *J Biol Chem* 2001, **276:** 29382-29392.

64. Morello JP, Salahpour A, Petaja-Repo UE, Laperriere A, Lonergan M, Arthus MF: **Association of calnexin with wild type and mutant AVPR2 that causes nephrogenic diabetes insipidus.** *Biochemistry* 2001, **40:** 6766-6775.

65. Pasel K, Schulz A, Timmermann K, Linnemann K, Hoeltzenbein M, Jaaskelainen J: **Functional characterization of the molecular defects causing nephrogenic diabetes insipidus in eight families.** *J Clin Endocrinol Metab* 2000, **85:** 1703-1710.

66. Morello JP, Salahpour A, Laperriere A, Bernier V, Arthus MF, Lonergan M: **Pharmacological chaperones rescue cell-surface expression and function of misfolded V2 vasopressin receptor mutants.** *J Clin Invest* 2000, **105:** 887-895.

67. Oakley RH, Laporte SA, Holt JA, Barak LS, Caron MG: **Association of beta-arrestin with G protein-coupled receptors during clathrin-mediated endocytosis dictates the profile of receptor resensitization.** *J Biol Chem* 1999, **274:** 32248-32257.
